# Early Anal Protrusion of Distal Ventriculoperitoneal Catheter Due to Iatrogenic Colonic Perforation: A Case Report and Review of Literature

**DOI:** 10.7759/cureus.20296

**Published:** 2021-12-09

**Authors:** Hattan H Bosy, Bushra M Albarnawi, Khalid M Ashour, Afnan Alyasi, Amjad S Alsulaihebi

**Affiliations:** 1 Medicine and Surgery, Umm AlQura University, Makkah, SAU; 2 Medicine and Surgery, Umm Al-Qura University, Makkah, SAU; 3 Neurological Surgery, Alexandria University, Alexandria, EGY; 4 Neurosurgery, Alnoor Specialist Hospital, Holy Mecca, SAU

**Keywords:** peritoneal chatheter, shunt complications., anal protrusion, bowel perforation, iatrogenic colonic perforation, hydrocephalus, shunt distal catheter, vp shunt, ventriculo-peritoneal shunt

## Abstract

Ventriculo-peritoneal (VP) shunt is one of the most commonly used therapeutic methods for hydrocephalus. And the incidence across the world of VP shunt complications varies from 20% to 45%. One of the rare complications is the catheter perforation of the abdominal viscera and its extrusion through the anal cavity. For the first time in the literature, this report addresses the presentation of iatrogenic perforation of the colon while inserting the peritoneal catheter of VP shunt.

Here, we present the case of a 15-year-old boy who is known to have cerebral palsy and congenital hydrocephalus with a VP shunt since birth. He presented to the ER with a history of headache and episodes of vomiting and was diagnosed with VP shunt dysfunction. The patient was taken for an emergency operation for shunt revision and a new shunt was placed. Then the next day he developed signs and symptoms of peritonitis with the distal part of the catheter protruding from the anal cavity, the patient was taken to the operating room for an exploratory laparotomy, the distal catheter was removed and replaced with external ventricular drainage, intra-operatively it was shown that the catheter was inserted directly into the colon causing bowel perforation, This report raises important questions about the nature of VP shunt bowel perforation and discusses management options.

## Introduction

Hydrocephalus is a fairly common disease, especially in children [1]. Ventriculo-peritoneal (VP) shunt is one of the most commonly used therapeutic methods for hydrocephalus, where the cerebrospinal fluid (CSF) from the lateral ventricle is typically drained into the peritoneal cavity. Other distal locations, such as the right atrium and the pleural cavity, are utilized occasionally [2]. Alternatively, endoscopic third ventriculostomy and choroid plexus cauterization can be used; Nonetheless, VP shunt is still the standard of care [1].

The overall incidence of VP shunt complications varies from 20% to 45% [3]. The most common potential complications include shunt obstruction, disconnection, infections, malposition, and seizures [4]. Subdural collection, abdominal hernia, abdominal CSF collection (pseudocyst), and catheter perforation of abdominal viscera and extrusion through the anal cavity are rare complications [5].

This paper sheds light on the unusual early-onset complication of VP shunts that to our knowledge is reported for the first time in the literature, which establishes the urgent need to address the presentation of iatrogenic perforation of the colon while inserting the peritoneal catheter of VP shunt.

## Case presentation

**Figure 1:**
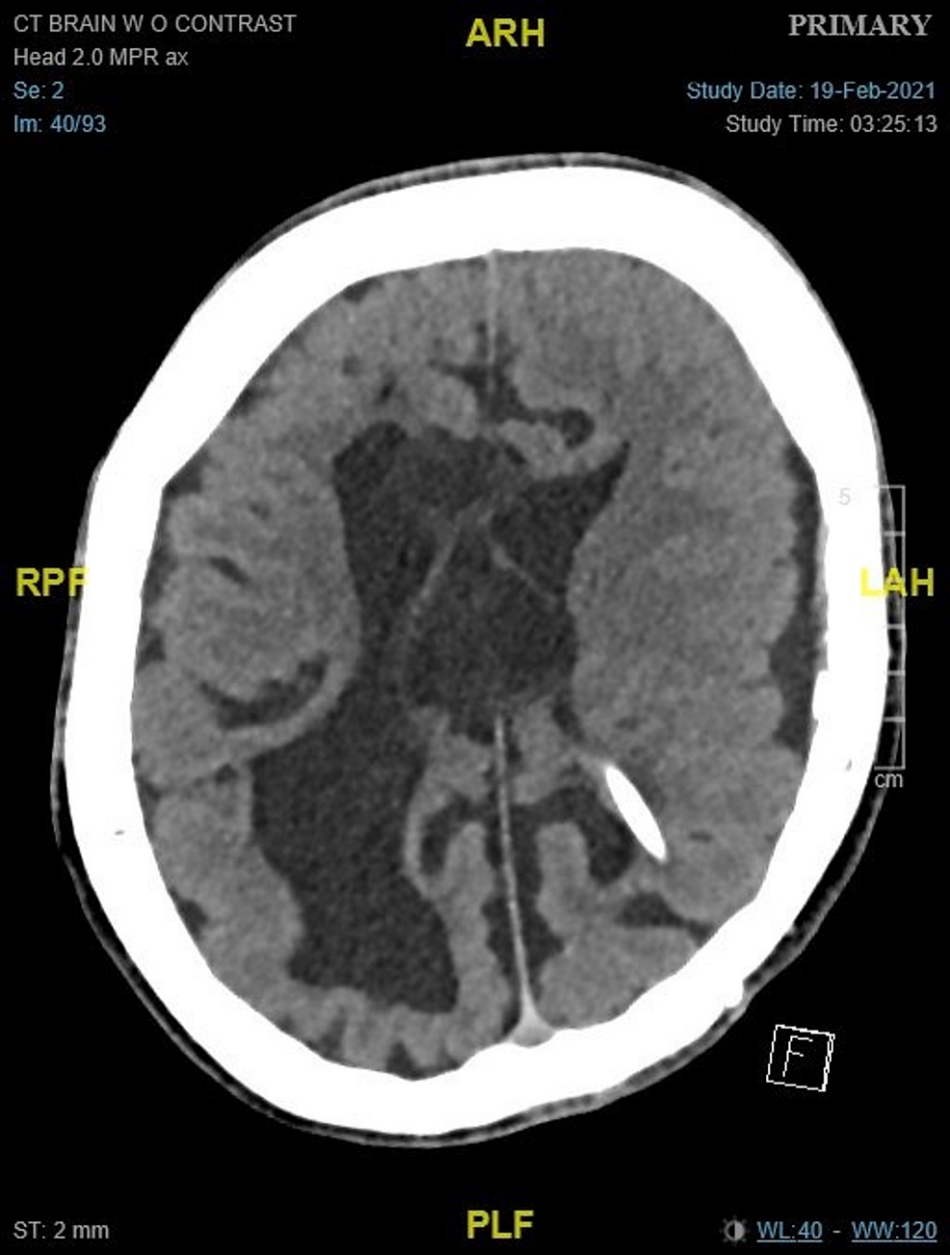
CT brain axial cut was done a few days before the patient's deterioration, showing the ventricular catheter in the left lateral ventricle and lax brain.

**Figure 2:**
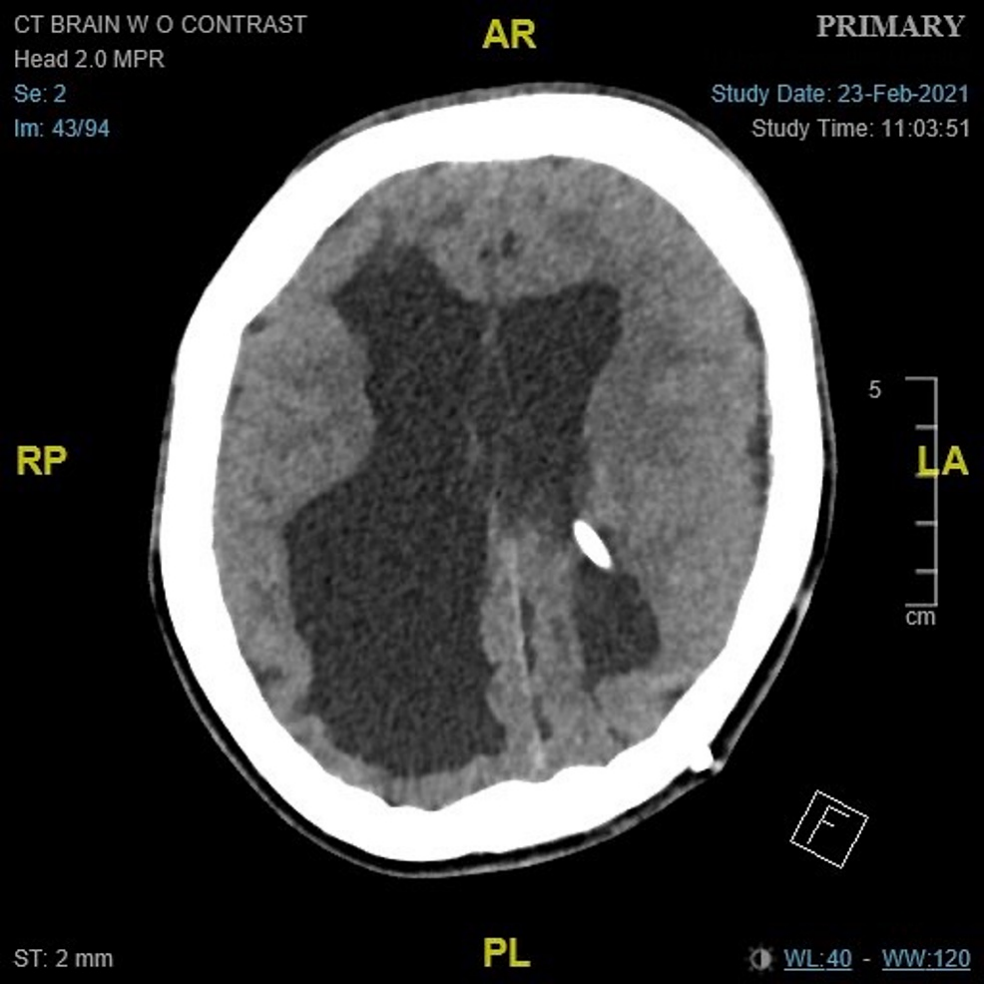
CT brain axial cut at the time of presentation in ER shows ventricular dilatation and periventricular lucency.

This is a 15-year-old boy. He is a case of cerebral palsy known to have VP shunt for congenital hydrocephalus since birth. He presented to the emergency department with a history of headaches and multiple episodes of vomiting for two days. On examination, he was conscious and communicating well with his mother with signs of dehydration due to the vomiting. The reservoir showed much-delayed refiling which indicates proximal obstruction. Computed tomography (CT) of the brain showed ventricular dilatation with periventricular lucency which confirmed VP shunt dysfunction (Figures 1, 2). An abdominal X-ray (AXR) revealed normal looping of a distal catheter inside the peritoneal cavity.

**Figure 3:**
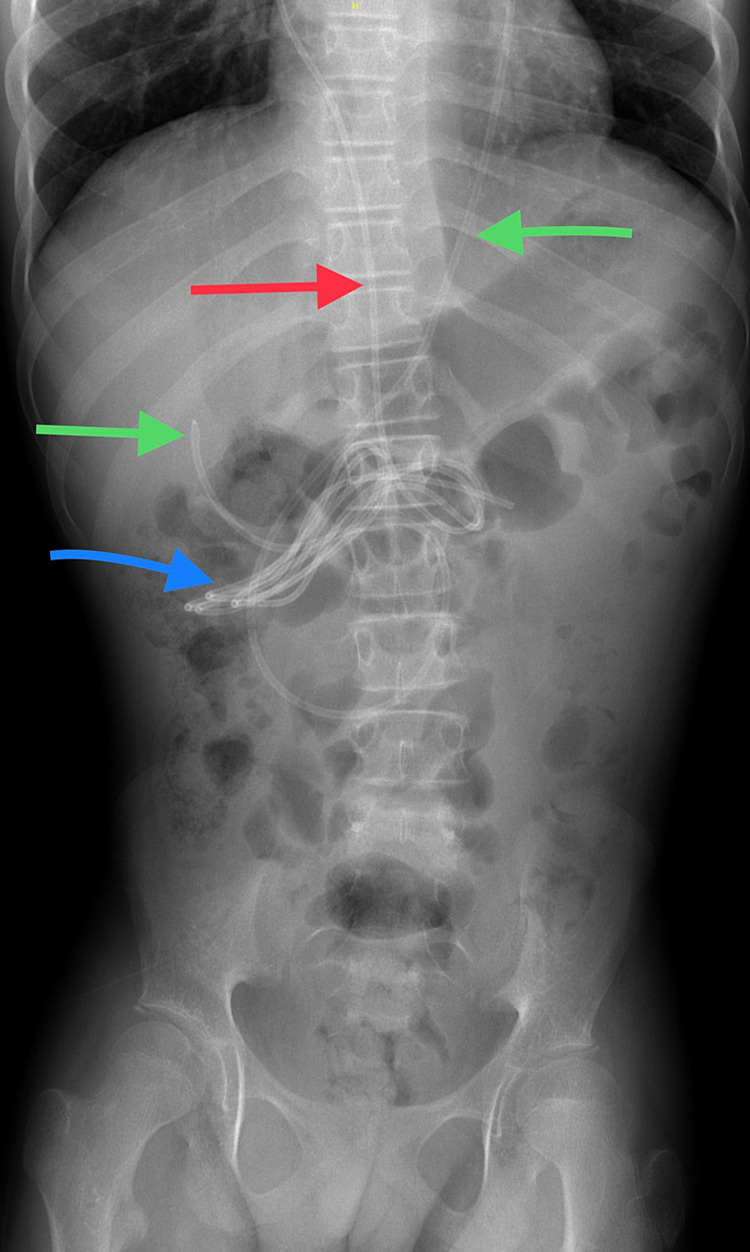
X-ray abdomen AP view - the red arrow refers to the new distal catheter, the green arrows refer to the old distal catheter, and the blue arrow refers to an abnormal multiple transverse looping of the new catheter at the transverse colon.

**Figure 4:**
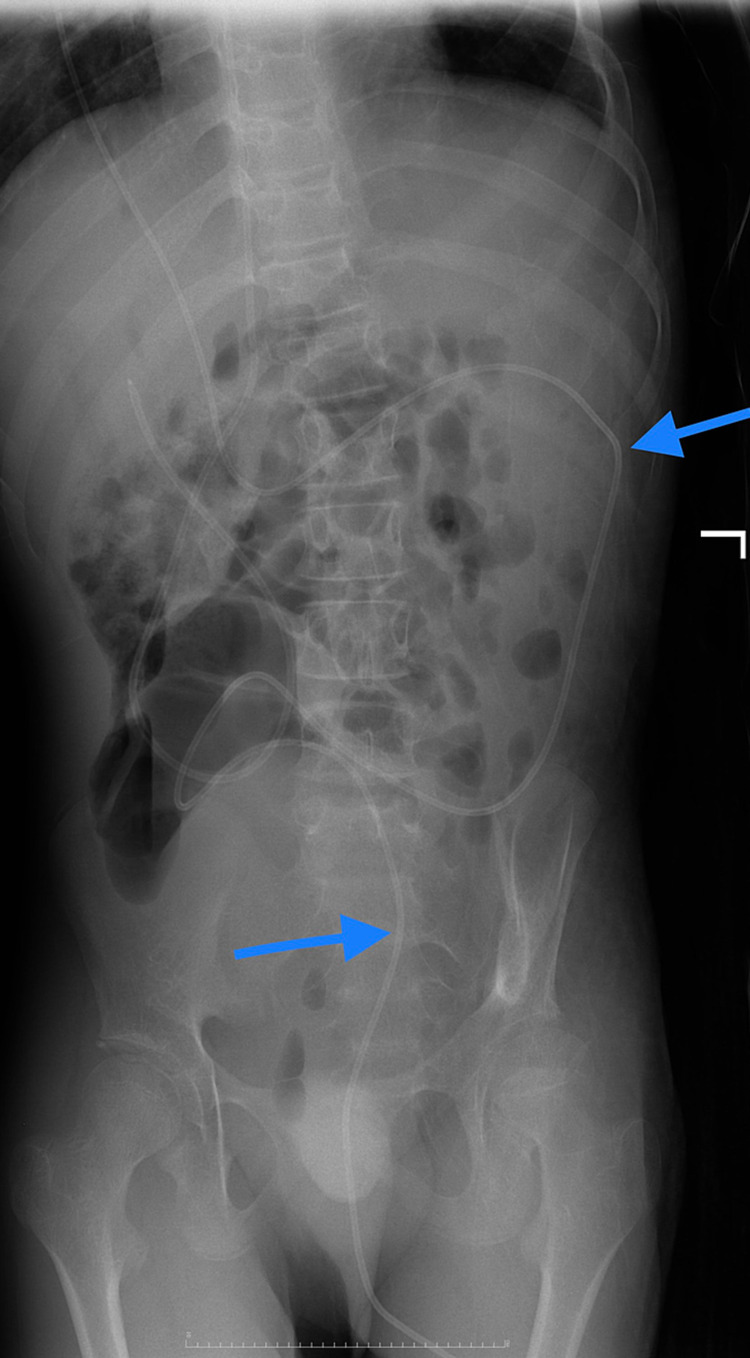
X-ray abdomen AP view - the blue arrows refer to the course of the catheter from the transverse colon down to the sigmoid colon, rectum, and anal canal.

The patient was taken for an emergency operation for shunt revision. A new shunt was inserted by an expert neurosurgeon who has been doing shunt operations for 15 years. The new distal catheter was inserted through a classical paramedian transverse mini-laparotomy incision, made on the same side of the old mini-laparotomy and 5 cm caudal to it. Then it was noticed that there were multiple abdominal adhesions and the peritoneum was thicker than normal. The old distal catheter was left as it was stuck to the abdomen. The next morning, the patient complained of abdominal pain and distention. He did not pass feces or flatus. On examination, there was abdominal tenderness and guarding. AXR showed abnormal transverse looping of the distal catheter at the epigastrium (Figure 3). By afternoon time, the distal catheter was seen out of the anal cavity. A new X-ray abdomen and pelvis showed the course of the catheter down from the transverse colon through the sigmoid colon and rectum (Figure 4). The VP shunt was converted to external ventricular drainage (EVD); the general surgeon found the colon tethered anteriorly to the abdominal wall at the site of the old scar, and the catheter was directly inserted into the colon. The distal part of the catheter was pulled out of the anal canal, after its proximal disconnection, and the bowel was eventually repaired.

A few days later, the patient started to have fecal discharge from the laparotomy incision necessitating an immediate intervention by the general surgeon to convert the repaired bowel to a colostomy.

The patient’s EVD was replaced multiple times during admission due to CSF infections. Once the infection was controlled, the EVD was replaced by a ventriculoatrial (VA) shunt. At the time of writing this case report, roughly nine months after surgery, the patient is in a good condition, still following up with general surgery, still on colostomy and his VA shunt is working well.

## Discussion

VP shunt obstruction - also known as malfunction - usually occurs proximally. Occasionally, it may occur distally or within the valve of the catheter [6]. The proximal part of the catheter may become blocked by brain matter or by pieces of the choroid plexus; another hypothesis to explain proximal obstruction suggests that materials such as blood may accumulate over time within the catheter, and ultimately clog it [6]. In distal catheter obstruction, the presence of tear in the distal end of peritoneal catheters is suggested to be associated with dysfunction of shunts [6]. Bowel perforation with the migration of the distal catheter of the VP shunt through the anal cavity is a rare but well-established complication that is categorized by Allouh et al. under the type of compound migration [7]. Shunt complications, such as distal catheter perforation of viscera in the abdomen, can develop over time [6-8]. Perforation of the colon by the distal catheter is a fairly rare complication that occurs at a rate of 0.1% to 0.7 % but has a high mortality rate of around 15% that is related to peritonitis, ventriculitis, or meningitis [9,10]. It can take place anytime ranging from weeks to several years after the shunt surgery and affects children more than adults [9-11]. The colon happens to be the most commonly reported segment of perforation, while it can occur at any site of the GI tract [11].

The etiology of bowel perforation due to the peritoneal catheter remains unclear in most cases. However, several risk factors and hypotheses have been suggested. Many researchers have proposed the formation of fibrosis that encases part of the catheter. This fibrosis is suspected to cause some pressure on the distal catheter, resulting in that area of the bowel being ulcerated, which eventually leads to bowel perforation [12]. It is also suggested that this mechanism of perforation is mainly caused by repetitive mechanical irritation by the stiff distal catheter to the small bowel due to peristalsis [12]. In a reported case by Bakal, adhesions have been noticed in between the colon and the distal catheter at or near the site of perforation [13]. Since the infant in that mentioned case had no prior history of any infection in the peritoneal cavity, the first operation of shunt insertion likely has resulted in these adhesions. It has also been suggested that children with congenital conditions such as myelomeningocele and congenital hydrocephalus could be more prone to developing perforation of the bowel due to friable bowel wall possibly caused by the deficient innervation [9-13]. Also, it has been demonstrated that catheters may cause allergies to people with silicone allergies and cause perforation with intestinal irritation and adhesion. Few cases of VP shunt-induced bowel perforation due to silicone allergy from the catheter have been mentioned in the literature [13].

Intra-abdominal adhesions between the scar in the anterior part of the abdominal wall and underlying visceral tissues are a common consequence of laparotomy. When adhesions form, the intestines become partially immobilized and are no longer able to move freely because they become tethered to the abdominal wall, to each other, or to other organs in the abdomen, which increases the risk of perforation during catheter insertion [14]. We believe that this is the major risk factor that contributes to this uncommon complication in the presented case. However, in reviewing the literature, no data were found on the association between a new site for distal catheter insertion and abdominal complications rate, a further study with more focus on location and technique is therefore suggested.

Prior studies have shown the superiority of the laparoscopic approach to open laparotomy for distal catheter insertion especially in cases with previous abdominal surgeries since the laparoscopic method allows for better control of the distal catheter location as it is under direct vision and it allows for easier navigation through the adhesions compared to the limited exposure in the mini-laparotomy approach. This allows the insertion to be done with fewer failed procedures and has a lesser complication rate [15,16]. However, this necessitates the collaboration of pediatric or general surgeons. 

When bowel perforation develops, the patient is usually asymptomatic. However, protrusion of the tip of the distal catheter from the anal canal is the most frequent presentation of symptomatic bowel perforation. About 15% to 25% or less of documented cases diagnosed with intestinal perforation also had signs of peritonitis, while 43% to 48% either developed ventriculitis or meningitis [9-17].

The catheter can be removed and managed using a variety of methods. Removal can be done manually by pulling the catheter through the anal canal after proximal disconnection if there is no intra-abdominal infection and no resistance is encountered, or via an upper or lower GI endoscopy, if the catheter was located more proximally or distally in the bowel, or surgically in case of severe intra-abdominal infection, either laparoscopically or through laparotomy as this allows the catheter to be removed while also closing the perforation site. The most appropriate method is usually determined by the patient's clinical condition [17].

## Conclusions

The purpose of presenting this report is to shed light on the possibility of the occurrence of this rare complication in the early or late stages of the postoperative period. As it is still fairly unknown as to what mainly leads to such complications, it is of great value to emphasize and report cases with unique presentations to such uncommon complications; which might help in putting forth more theories behind why they occur. In our case, we believe a reasonable future consideration could be as simple as making the new mini-laparotomy incision for distal catheter insertion as away as possible from any old abdominal scar, as this might play a role in avoiding similar complications due to old adhesions of the previous abdominal surgery. As well as considering the chronic conditions of the patient beforehand and accounting for anomalies that could come up during the catheter insertion, such as a friable colon that is liable to perforation. Such simple considerations might save the patient from devastating complications and unnecessary interventions.
